# Machine Learning-Based Multi-Objective Composition Optimization of High-Nitrogen Austenitic Stainless Steels

**DOI:** 10.3390/ma18235460

**Published:** 2025-12-03

**Authors:** Yinghu Wang, Long Chen, Limei Cheng, Enuo Wang, Zhendong Sheng, Ligang Zhang

**Affiliations:** 1 National Center for Materials Service Safety, University of Science and Technology Beijing, Beijing 100083, China; hihihowareyou@163.com; 2 Chengdu Institute of Advanced Metallic Material Technology and Industry Co., Ltd., Chengdu 610300, China; chenglimei_ustb@163.com (L.C.); panpan_wen@163.com (E.W.); shengzhendong@gmail.com (Z.S.); 3 School of Materials Science and Engineering, Central South University, Changsha 410083, China; cllong@csu.edu.cn

**Keywords:** machine learning, high-nitrogen austenitic stainless steel, multi-objective genetic optimization, thermodynamic calculation of phase diagrams

## Abstract

High-nitrogen austenitic stainless steels (HNASS) require compositional strategies that simultaneously maximize corrosion resistance and microstructural stability while suppressing delta (δ) ferrite and deleterious precipitates. Here, an explainable multi-objective design workflow is developed that couples thermodynamic descriptors from the Calculation of Phase Diagrams (CALPHAD) approach—using both equilibrium and Scheil solidification calculations—with machine learning surrogate models, random forest (RF) and Extreme Gradient Boosting (XGBoost), trained on 60,480 compositions in the Fe–C–N–Cr–Mn–Mo–Ni–Si space. The physics-informed feature set comprises phase fractions; transformation and precipitation temperatures for δ-ferrite, chromium nitride (Cr_2_N), sigma (σ) phase and M_23_C_6_ carbides; liquidus and solidus temperatures; and the pitting-resistance equivalent number (PREN). The RF model achieves consistently low prediction errors, with a PREN root-mean-square error (RMSE) of ≈0.004, and exhibits strong generalization. Shapley additive explanations (SHAP) reveal metallurgically consistent trends: increasing nitrogen (N) suppresses δ-ferrite and promotes Cr_2_N; carbon (C) promotes M_23_C_6_; molybdenum (Mo) promotes the σ-phase; and C and silicon (Si) widen the freezing range. Using the trained surrogate as the objective evaluator, the non-dominated sorting genetic algorithm III (NSGA-III) builds Pareto fronts that minimize the δ-ferrite range, Cr_2_N, σ-phase, M_23_C_6_ and the freezing range (ΔT) while maximizing PREN. The Technique for Order Preference by Similarity to Ideal Solution (TOPSIS) is then applied to rank the Pareto-optimal candidates and to select compositions that combine elevated PREN with controlled precipitation windows. This workflow is efficient, reproducible and interpretable and provides actionable composition candidates together with a transferable methodology for data-driven stainless steel design.

## 1. Introduction

High-nitrogen austenitic stainless steels (HNASS) have emerged as a new generation of high-performance structural materials owing to their outstanding combination of corrosion resistance and mechanical properties in demanding service environments [1,2,3,4]. With a stable and homogeneous austenitic microstructure, HNASS are widely used in protective armor, shipbuilding, marine engineering and the chemical industry [5,6,7,8,9,10]. The solid-solution strengthening provided by nitrogen significantly increases the yield strength and hardness of HNASS. Moreover, nitrogen stabilizes the austenitic phase and enhances resistance to pitting and crevice corrosion [11,12,13], making these alloys particularly attractive for structural components that must withstand both high stresses and aggressive media. Benefiting from this property portfolio, HNASS are promising alternatives to conventional austenitic stainless steels, especially for applications under extreme conditions such as high pressure, elevated temperature and highly corrosive solutions. Therefore, systematic investigation of compositional design, microstructural control and performance evolution in HNASS is of considerable scientific and engineering significance. However, the limited solubility of nitrogen in molten steel, the propensity for nitride formation during solidification and the difficulty of precisely controlling nitrogen content during melting or additive manufacturing remain key challenges that hinder large-scale industrial deployment [14].

Although nitrogen is a minor alloying element, its influence on the tensile behavior [15,16,17] and fatigue performance [18,19,20] of steels has been extensively investigated. Nitrogen markedly increases fatigue life by promoting planar slip and inducing cyclic softening in the early stages of damage, thereby enhancing fatigue strength and reducing plastic strain accumulation; together, these effects delay crack initiation and retard subsequent crack growth [21,22,23]. Nitrogen additions also stabilize austenite and broaden the composition and temperature ranges of the austenitic phase, thereby suppressing ferrite formation [24,25,26]. In addition, nitrogen refines the austenitic grain structure [27,28,29] and improves the mechanical properties of austenitic stainless steels. From a corrosion perspective, nitrogen enhances localized corrosion resistance—particularly resistance to pitting and intergranular attack. For example, Gao et al. [30] examined the effect of nitrogen content on the composition, structure and protective performance of passive films in HNASS exposed to 0.5 mol L^−1^ NaCl. Alloys with higher nitrogen contents exhibited a greater fraction of low-angle grain boundaries, lower corrosion current density and a reduced corrosion rate. The formation of stable oxides such as Cr_2_O_3_ in the passive film facilitates passivation and repassivation, underpinning the superior corrosion resistance of HNASS. Despite these advantages over conventional austenitic grades, the rate-dependent mechanical response of HNASS and the associated microstructural evolution remain less explored. Zhang et al. [31] systematically investigated the dynamic mechanical properties and microstructural evolution of HNASS over a wide strain-rate range, providing important insights but also highlighting the need for further mechanistic studies.

Despite these favorable properties, the compositional design of HNASS still faces significant challenges, primarily due to the complex coupling among nitrogen solubility, phase stability and corrosion resistance. Traditional trial-and-error alloy development and single-objective optimization are inefficient and often fail to capture nonlinear interactions among multiple alloying elements. Such empirical design approaches rely solely on experimental data and tend to overlook the underlying physical and chemical mechanisms. Furthermore, repeated alloy synthesis and testing make the design process time-consuming and costly. 

To address these challenges, researchers have increasingly combined thermodynamic calculations, alloy design and data-driven optimization to tailor the composition and processing parameters of HNASS. Thermodynamic modeling based on the CALPHAD approach enables accurate prediction of phase stability, solubility limits and precipitation behavior in Fe–Cr–Mn–N–C–Si systems [32,33,34,35], and these predictions have been corroborated by experimental validations of phase equilibria and nitrogen solubility [36,37,38]. In parallel, machine learning (ML) has emerged as a powerful tool for materials optimization, efficiently exploring high-dimensional design spaces [39,40,41,42]. ML-assisted CALPHAD frameworks have been successfully applied to optimize microstructures and mechanical properties in steels, superalloys and titanium alloys, markedly improving prediction accuracy and computational efficiency [43,44,45]. In the context of high-nitrogen austenitic systems, Khan et al. [46] employed an extremely randomized trees (ETR) model to predict the yield strength and ultimate tensile strength of Cr–Mn–N alloys. Ahmadi et al. [47] developed a back-propagation artificial neural network (BP-ANN) to predict the high-temperature flow stress of API 5CT-L80 steel, achieving a high correlation coefficient and demonstrating the strong predictive capability of the BP-ANN model. Similarly, Wang et al. [48] constructed processing maps for P550 steel using a data-driven approach, in which flow-stress data generated by an ANN were used to build the maps and were subsequently validated experimentally, confirming the reliability of ANN-based process design. 

Nevertheless, several critical scientific issues remain unresolved: (i) discrepancies between thermodynamic predictions and experimental observations under non-equilibrium solidification and rapid cooling; (ii) largely empirical selection of features when constructing composition–property relationships; (iii) the predominance of single-objective machine learning optimization that overlooks trade-offs between microstructural stability and corrosion resistance; and (iv) limited transferability and generalization of current models across alloy systems and processing routes. These limitations hinder quantitative and accurate compositional design of HNASS for engineering applications. 

In view of the above, this study proposes an explainable, multi-objective alloy-design framework that integrates CALPHAD-derived thermodynamic data with machine learning algorithms to achieve composition–property optimization for HNASS. Thermodynamic descriptors—including liquidus and solidus temperatures, phase-transformation ranges and Gibbs energies—are extracted from the Fe–Cr–Mn–Ni–N–C–Si system via CALPHAD simulations. Feature-selection methods are then employed to identify the key factors governing microstructural stability and corrosion resistance. Subsequently, the trained surrogate is coupled with a multi-objective genetic algorithm, specifically the non-dominated sorting genetic algorithm III (NSGA-III) [49], to generate Pareto-optimal compositions that balance phase-stability descriptors and corrosion resistance.

This framework establishes a hybrid, theory- and data-driven pathway for intelligent compositional design of HNASS and provides a transferable methodology for developing next-generation stainless steels with superior performance and processability.

## 2. Materials and Methods

### 2.1. HTC Calculation

**Figure 1:**
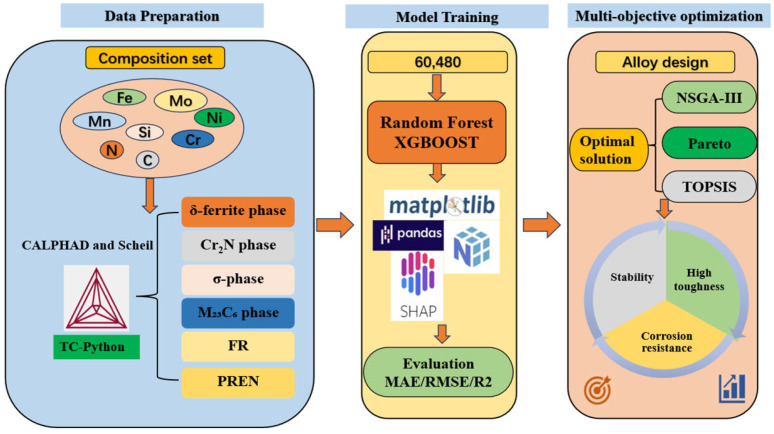
Alloy composition design strategy.

Thermodynamic calculations were performed within the CALPHAD framework using Thermo-Calc^®^ 2021b with the TCFE11 database, which covers the relevant binary and multicomponent systems over the composition and temperature ranges pertinent to the target HNASS. The alloy design sought to balance mechanical stability and corrosion resistance while preserving austenite stability. Manganese (Mn) and nitrogen (N) were introduced to partially replace nickel (Ni), thereby stabilizing the austenitic phase and enhancing mechanical performance. Candidate compositions were selected with reference to the literature and commercial alloy specifications. Figure 1 summarizes the overall alloy composition design strategy and the associated thermodynamic–data-driven workflow.

First, the compositional domain was defined from commercially available HNASS summarized in Ref. [50]. Around these baseline alloys, the ranges of C, N, Cr, Mn, Mo, Ni and Si were extrapolated to construct an extended design space (Table 1). Each element was discretized into a finite number of levels (e.g., six levels for N, five for Mo, six for Cr and Ni, seven for Mn, two for Si and four for C), and all combinations within the specified ranges were enumerated, yielding 60,480 candidate compositions. Subsequently, thermodynamic equilibrium calculations and Scheil solidification simulations were performed to obtain, for each composition, phase fractions, transformation/precipitation temperatures and solid–liquid boundaries (liquidus and solidus).

The precipitation of sigma (σ) phase—a Cr- and Mo-rich intermetallic compound—significantly deteriorates the toughness of high-nitrogen stainless steels, while excessive carbide formation and the presence of delta (δ) ferrite also impair corrosion resistance and mechanical properties. Therefore, the following thermodynamic criteria were imposed in the present alloy design: (i) a wide single-phase austenite field must be present; (ii) the stability windows of σ and carbide phases should be restricted—no σ at 750 °C and no carbides above 960 °C; and (iii) the δ-ferrite temperature range above 900 °C should be minimized. These thresholds were defined with reference to the equilibrium phase diagram of HNASS. This grid-based construction ensures that the initial sampling is uniform across the prescribed composition ranges, and any subsequent biases arise only from the thermodynamic and PREN-based filters rather than from the sampling procedure itself.

To further enhance the pitting resistance of high-nitrogen austenitic stainless steels and refine the candidate set, the pitting resistance equivalent number (PREN) was used as an additional screening descriptor. PREN accounts for the beneficial contributions of Cr and Mo, and its modified form ${PREN}_{mod}$ = Cr + 3.3Mo + 16N [51] explicitly incorporates the strong effect of nitrogen. Compositions with higher PREN values were preferentially retained for subsequent modeling and optimization.

**Figure 2:**
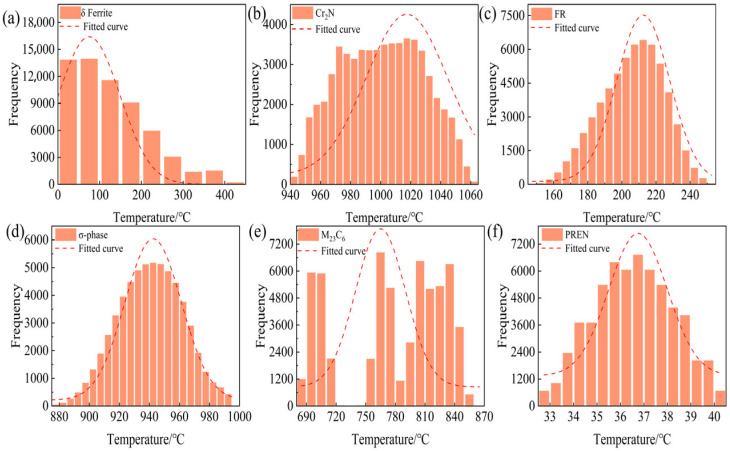
Histograms of the six target descriptors for the 60,480 candidate high-nitrogen austenitic stainless steel compositions: (**a**) δ-ferrite temperature range, (**b**) Cr_2_N precipitation/dissolution temperature, (**c**) freezing range ΔT, (**d**) σ-phase precipitation temperature, (**e**) M_23_C_6_ precipitation temperature and (**f**) PREN. Bars represent the frequency of CALPHAD-predicted values, and the dashed curves indicate the corresponding fitted probability density distributions.

Figure 2 summarizes the distributions of six descriptors. The δ-ferrite-related temperatures are strongly right-skewed, with a primary mode at 50–120 °C. The freezing range is unimodal, peaking at ≈180–220 °C with slight right skew. Cr_2_N precipitation temperatures cluster narrowly between 1000 and 1035 °C. The σ-phase sensitivity window centers at 960–970 °C and is approximately normal. M_23_C_6_ exhibits a clearly multimodal pattern with secondary modes near 780 °C and 840 °C. PREN is near-normally distributed and concentrated at 36–38 (dimensionless). Overall, the set displays both skewness and multimodality.

These characteristics motivate the use of distribution-robust, tree-based models (e.g., random forest (RF) and XGBoost version 2.1.4), together with stratified sampling based on descriptor bins and, where appropriate, monotonic transformations (e.g., the Yeo–Johnson transformation) for the δ-ferrite and M_23_C_6_ targets. For downstream optimization, the objectives are defined to minimize the freezing range ΔT(FR) and the phase-formation temperatures of δ-ferrite, Cr_2_N, σ-phase and M_23_C_6_ while maintaining a high PREN and avoiding the sensitivity windows associated with σ, Cr_2_N and M_23_C_6_.

### 2.2. Machine Learning Model

To accurately characterize the complex, nonlinear relationships among chemical composition, thermodynamic descriptors and performance metrics (e.g., corrosion resistance and phase stability), we employ two supervised learning algorithms—RF and Extreme Gradient Boosting (XGBoost)—to model data generated by thermodynamic simulations. RF is an ensemble method that constructs multiple decorrelated decision trees and averages their predictions, thereby improving generalization and mitigating overfitting, particularly in the presence of strong nonlinearities and high-dimensional feature interactions. XGBoost, a gradient-boosted decision tree algorithm, builds trees sequentially to minimize a regularized objective; its shrinkage, subsampling, and L1/L2 penalties provide effective bias–variance control and state-of-the-art predictive accuracy on tabular, heterogeneous data.

XGBoost is an efficient implementation of the gradient boosting decision tree (GBDT) framework. It trains an ensemble of weak learners sequentially by following the (approximate) negative gradient of a specified loss function, with each tree fitting the residuals left by the current ensemble. XGBoost delivers high predictive accuracy and training efficiency on large-scale datasets with sparse and nonlinearly coupled features. Its regularization (L1/L2 penalties), shrinkage (learning rate) and row/column subsampling—together with robust handling of missing values—mitigate overfitting and enhance generalization.

RF and Extreme Gradient Boosting (XGBoost) are complementary tree-based ensemble learners: RF implements bootstrap aggregation (bagging) of decorrelated decision trees, whereas XGBoost implements gradient-boosted decision trees (GBDT) [52,53,54]. Accordingly, we employ RF and XGBoost to train predictive models on the thermodynamics-derived dataset and to evaluate their accuracy and robustness.

### 2.3. Model Performance Metrics

To evaluate model performance, we used the root-mean-square error (*RMSE*), mean absolute error (*MAE*), coefficient of determination (*R*^2^) and Pearson correlation coefficient (*r*) as performance metrics, computed as Equations (1)–(4) [55,56].

(1) $$RMSE=\sqrt{\frac{1}{n}\sum_{i=1}^{n}{\left(y_{i}-x_{i}\right)}^{2}}$$

(2) $$MAE=\frac{1}{n}\sum_{i=1}^{n}\left|y_{i}-x_{i}\right|$$

(3) $$R^{2}=1-\frac{{\sum_{i=1}^{n}\left(y_{i}-x_{i}\right)}^{2}}{{\sum_{i=1}^{n}\left(\overset{ˉ}{y}-x_{i}\right)}^{2}}$$

(4) $$r=\frac{\sum_{i=1}^{n}\left(x_{i}-\overset{ˉ}{x}\right)\left(y_{i}-\overset{ˉ}{y}\right)}{\sqrt{{\sum_{i=1}^{n}\left(x_{i}-\overset{ˉ}{x}\right)}^{2}}\sqrt{{\sum_{i=1}^{n}\left(y_{i}-\overset{ˉ}{y}\right)}^{2}}}$$

where $x_{i}$ denotes the CALPHAD-derived target value (e.g., transformation temperature or PREN) for the *i*-th composition, $\overset{ˉ}{x}$ is the arithmetic mean of all CALPHAD target values in the evaluation set, $y_{i}$ denotes the corresponding value predicted by the surrogate model, $\overset{ˉ}{y}$ is the mean of all predicted values, and *n* is the total number of samples in the evaluation set.

## 3. Results and Discussion

### 3.1. Correlation Analysis

**Figure 3:**
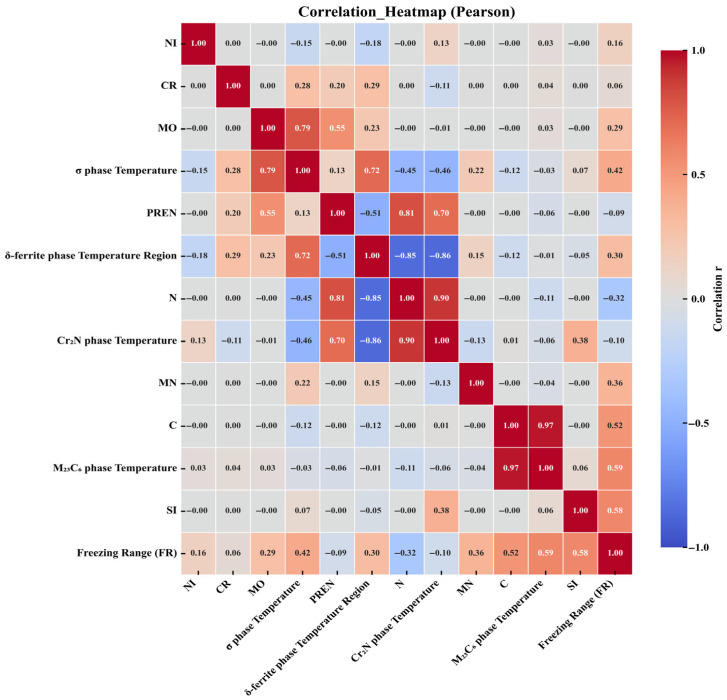
Correlation plot between molecular descriptors.

In this study, six scalar objectives—δ-ferrite temperature range, Cr_2_N precipitation/dissolution temperature, σ-phase precipitation temperature, M_23_C_6_ precipitation temperature, PREN and the freezing range ΔT—are considered as functions of a common set of compositional features (C, N, Cr, Mn, Mo, Ni, Si). Pearson correlation analysis reveals several strong linear relationships (Figure 3). Carbon exhibits a very strong positive correlation with the M_23_C_6_ precipitation temperature (r ≈ 0.97) and a moderate positive correlation with the freezing range (r ≈ 0.52), indicating that the carbide-forming tendency is aligned with both precipitation temperature and the liquidus–solidus span. Molybdenum correlates strongly with the σ-phase precipitation temperature (r ≈ 0.79) and moderately with PREN (r ≈ 0.55), whereas chromium shows only weak-to-moderate correlations (r ≈ 0.28–0.29) with selected transformation temperatures, including the σ-phase precipitation temperature and the δ-ferrite range. Nitrogen displays the opposite trend: it is strongly positively correlated with the Cr_2_N precipitation/dissolution temperature (r ≈ 0.90) and strongly negatively correlated with the δ-ferrite range (r ≈ −0.85); these two targets are themselves strongly negatively correlated (r ≈ −0.86), reflecting the antagonism between nitride formation and ferrite stability. PREN correlates strongly with N (r ≈ 0.81), moderately negatively with the δ-ferrite range (r ≈ −0.51), and moderately positively with Mo (r ≈ 0.55). Nickel shows near-zero first-order (linear) correlations with most targets, implying limited marginal effects within the studied domain. Overall, the correlation matrix exhibits a modular structure: (i) a positively correlated C–M_23_C_6_–Si–ΔT cluster; (ii) an N–Cr_2_N cluster that is negatively associated with the δ-ferrite range; and (iii) a Mo-centered cluster dominated by the σ-phase precipitation temperature.

It should be emphasized that this figure is based on Pearson correlation (Figure 3), which measures first-order linear relationships and cannot be used for causal inference; extreme values and nonlinear relationships may weaken or obscure correlation strength. Since clustering used ∣r∣ as the similarity measure, variables grouped together represent “high correlation” rather than “same direction,” and interpretation should also refer to the color (sign); Feature Description (Table 2). In this work, the input feature vector is restricted to the seven elemental mass fractions (C, N, Cr, Mn, Mo, Ni and Si), while the “physics-informed” aspect resides in the manual selection of thermodynamic target descriptors—δ-ferrite temperature range, Cr_2_N, σ-phase and M_23_C_6_ precipitation temperatures, the freezing range and PREN—based on established metallurgical knowledge of phase stability and corrosion resistance in HNASS. This choice was subsequently validated by the Pearson correlation and SHAP analyses in Section 3.1 and Section 3.3. As a sensitivity check, surrogate models retrained on reduced feature sets that excluded consistently low-importance elements exhibited only minor changes in R^2^ and RMSE, indicating that the predictions are not unduly sensitive to these weak contributors.

### 3.2. Evaluation of Different Models

This study adopts a multi-target regression strategy in which six scalar objectives—δ-ferrite temperature range, Cr_2_N precipitation temperature, σ-phase precipitation temperature, M_23_C_6_ precipitation temperature, PREN and the freezing range ΔT—are each modeled as functions of a common set of compositional features (C, N, Cr, Mn, Mo, Ni, Si). Tree-based ensemble learners, namely random forest (RF) and Extreme Gradient Boosting (XGBoost), are used as the core predictors. At the data-processing stage, any missing or non-finite feature values were imputed with the corresponding feature-wise mean. At the modeling stage, random seeds were fixed, the data were split into training and testing sets with an 80–20 ratio, and a one-model-per-target strategy was adopted to fit each objective separately. To obtain robust hyperparameters, a genetic algorithm was employed to optimize the XGBoost model by minimizing the three-fold cross-validation mean squared error; L1 and L2 regularizations (reg_alpha, reg_lambda), together with learning-rate shrinkage and subsampling, were used to mitigate overfitting. RF served as a parallel baseline and cross-validated comparator to assess the bias–variance trade-off. After training, RMSE and R^2^ were computed on both the training and testing sets to quantify goodness of fit and generalization. The trained models and their evaluation outputs were stored for reproducibility and for subsequent interpretation and optimization. Overall, the pipeline combines cross-validation-guided evolutionary hyperparameter tuning with ensemble learning under reproducible settings, yielding accurate and stable predictors for all six targets.

Under the same data-cleaning and train–test splitting protocol, the random forest (RF) model consistently outperforms XGBoost across all six regression targets. Table 3 and Table 4 summarize the performance metrics of the two machine learning models. For both the temperature-related objectives and the alloy corrosion-resistance indicator PREN, RF achieves lower RMSE and higher R^2^, with very similar training and testing scores, indicating good generalization. For example, the test R^2^ for the δ-ferrite precipitation temperature increases from 0.9113 with XGBoost to 0.9804 with RF, and the freezing range, Cr_2_N precipitation temperature and σ-phase precipitation temperature also show systematic improvements. For PREN, the RF model is nearly unbiased (RMSE ≈ 0.004, R^2^ ≈ 0.999), whereas XGBoost still exhibits noticeable errors (RMSE ≈ 0.3086, R^2^ ≈ 0.9659). These results suggest that, for the present feature–response relationships and hyperparameter choices, the bagging-based tree ensemble captures nonlinearities and interactions more effectively, while the gradient-boosting model would require further tuning (e.g., more weak learners, relaxed regularization and a broader hyperparameter search) to reach comparable performance.

**Figure 4:**
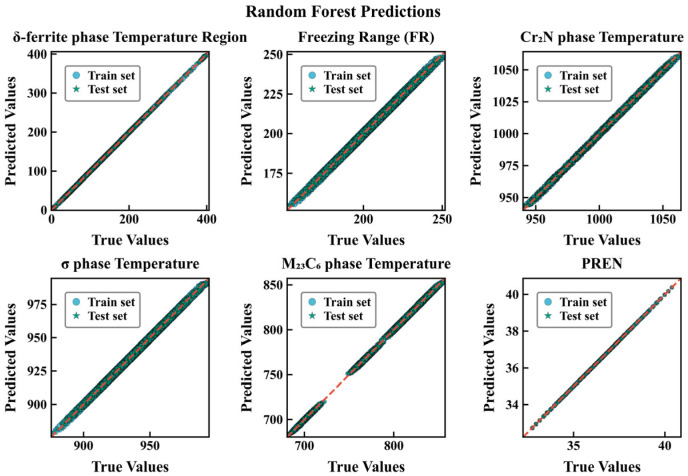
Predicted versus true values for six target properties using the RF regression model.

**Figure 5:**
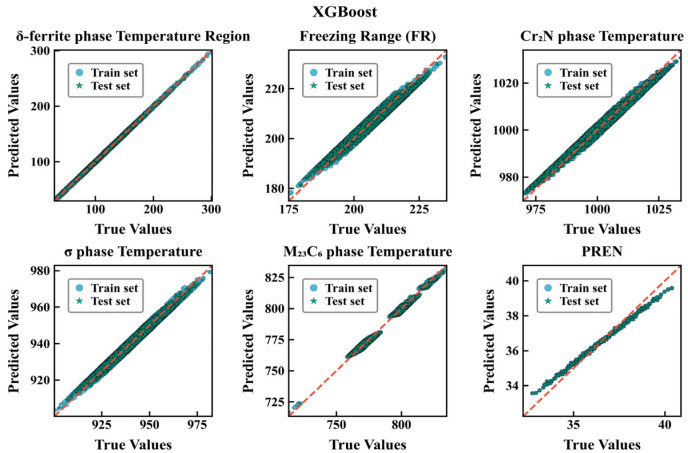
Predicted versus true values for six target properties using the XGBoost regression model.

To intuitively evaluate the fitting and generalization performance of the two types of models on each target, this paper plots prediction–true value comparison charts for RF and XGBoost (Figure 4, Figure 5), respectively. Each subplot uses equal-axis coordinates to display the predicted and true values of the test/training samples, with y = x as the reference baseline; a consistent Nature Publishing Group (NPG) color scheme is used to distinguish data subsets (circles for the training set, stars for the test set), with outer edge strokes to enhance recognizability. To improve readability of the scatter plots, points with absolute residuals exceeding a threshold of |y − ŷ| > 3 (in the respective physical units) are visually truncated. This heuristic choice corresponds to removing less than X% of the samples and serves only to reduce clutter; it does not affect the fitted models or the numerical error metrics reported in Table 3 and Table 4. The results show that for all targets, the scatter points of RF are more tightly clustered along y = x, with highly consistent training and test distributions; XGBoost shows more obvious dispersion and deviation in indicators such as δ-ferrite phase range precipitation temperature and FR. Combined with the aforementioned RMSE/R^2^ metrics (for example, PREN under RF is almost unbiased, R^2^ ≈ 1.000; whereas XGBoost still has visible errors), it can be considered that bagging-based ensembles are more robust to the current feature–response relationships and noise structure, exhibiting stronger generalization consistency. It should be emphasized that these performance metrics quantify how accurately the surrogates reproduce the CALPHAD-derived thermodynamic descriptors, rather than experimental measurements. The models are therefore intended as numerical surrogates for the underlying thermodynamic calculations.

### 3.3. SHAP Analysis

The SHAP (SHapley Additive exPlanations) value is a method for explaining model prediction results. It evaluates the importance of each feature by calculating the contribution of the feature to the model output. The essence of the SHAP value is a Shapley value, which originates from the concept of the Shapley value in game theory, used to measure each player’s contribution in a game.

In machine learning, SHAP values are used to explain the importance contribution of the model’s prediction results for each sample. Specifically, for each sample, the SHAP value tells us the degree to which each feature influences the model’s prediction for that sample, thereby explaining the model’s prediction results, helping to understand the model’s decision-making process, and discovering hidden patterns and trends in the data.

SHAP values can be used to explain the prediction results of a RF model, and the summary plot is a commonly used visualization method. The summary plot can intuitively show the contribution of each feature to the model’s prediction results and can also consider multiple features at the same time, helping us understand the model’s decision-making process. SHAP values provide insights into how each feature affects prediction outcomes by capturing both positive and negative effects across different samples [57,58].

**Figure 6:**
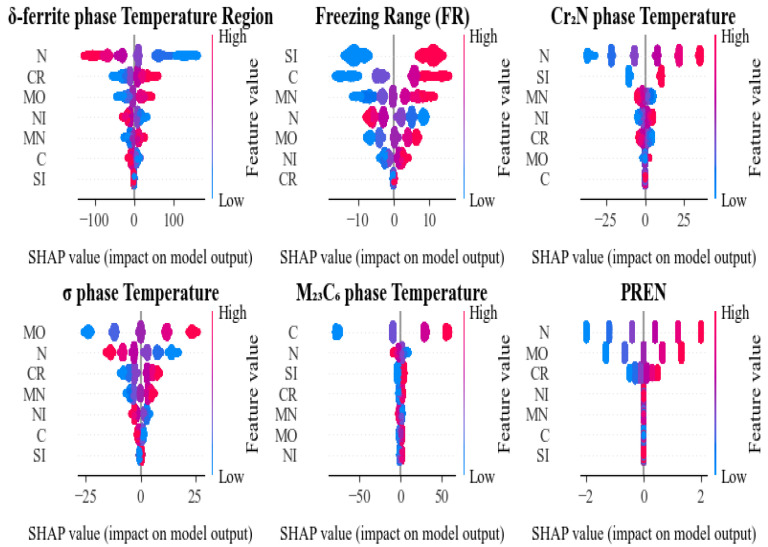
Model interpretation analyzed by SHAP.

To gain a deeper understanding of the impact of different component elements on the prediction results of various performance indicators, this study calculated Shapley Additive Explanation values (SHAP values) based on the RF model and plotted SHAP summary plots for each target variable (Figure 6). The horizontal axis represents the SHAP values, reflecting the direction and magnitude of a feature’s marginal contribution to the prediction output; the vertical axis represents the input features, and the color of the points indicates the feature value (blue for low values, red for high values). The greater the horizontal dispersion of the points, the stronger the influence of that feature on the model output.

First, in the prediction of the δ-ferrite phase temperature region, nitrogen (N) appears as the primary driving factor, with high N content showing a significant negative contribution to the predicted value, while chromium (Cr) and molybdenum (Mo) exhibit positive effects in certain ranges, indicating their important role in stabilizing phase transformation temperature.

**Figure 7:**
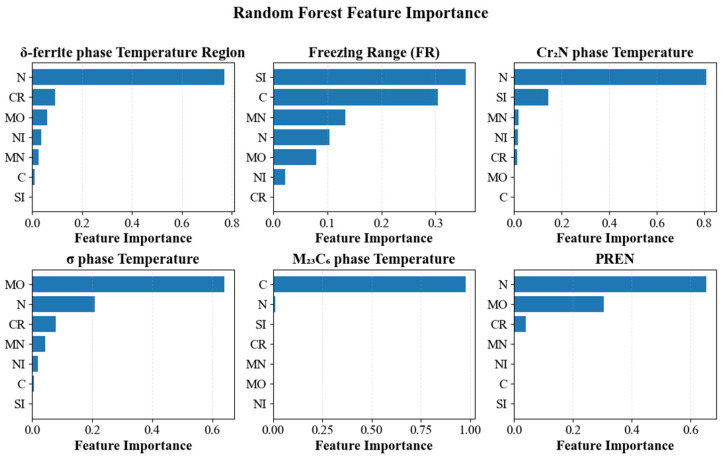
Random forest feature importance for six targets.

As shown in Figure 7, RF feature importances identify physically consistent drivers for the six targets. The δ-ferrite temperature range is dominated by N, with Cr and Mo as secondary contributors and Ni and Mn minor; C and Si are negligible. The freezing range is governed mainly by Si and C, with smaller effects from Mn and Mo. The Cr_2_N precipitation temperature is driven primarily by N. The σ-phase temperature is governed by Mo. The M_23_C_6_ precipitation temperature is controlled by C. PREN depends strongly on N and Mo, with a weaker contribution from Cr. These rankings accord with established metallurgy—N stabilizes austenite and promotes nitrides, Mo promotes σ, C governs carbide formation, and C and Si widen the liquidus–solidus interval—they are consistent with our SHAP and permutation analyses.

For the freezing range, silicon (Si) and carbon (C) emerge as the dominant predictors. High Si values are associated with negative SHAP contributions, indicating a narrowing of the freezing range, whereas high C values show positive SHAP contributions, indicating an expansion of the freezing range. Manganese (Mn) and nitrogen (N) provide moderate contributions. These results highlight the multi-element, synergistic regulation of solidification behavior and underscore the importance of considering coupled compositional effects when optimizing the freezing range.

For the Cr_2_N precipitation temperature, nitrogen (N) is the dominant predictor: higher N levels yield strongly positive SHAP values, indicating an increase in the predicted transformation temperature. In contrast, chromium (Cr) and molybdenum (Mo) exhibit negative contributions, lowering the predicted temperature within the examined composition range. This pattern suggests that elevated nitrogen activity stabilizes Cr_2_N, whereas Cr and Mo tend to depress its transformation temperature under the present conditions.

For the prediction of the σ-phase precipitation temperature, molybdenum (Mo) is the most influential feature: higher Mo levels produce strongly positive effects, indicating an increase in the predicted σ-phase precipitation temperature. Chromium (Cr) and PREN-related descriptors also contribute positively, albeit to a lesser extent. These trends are consistent with the metallurgical understanding that Mo and Cr promote σ-phase formation within the examined composition range.

For M_23_C_6_ phase, carbon (C) is the most critical contributing factor, with high carbon levels strongly promoting the precipitation of M_23_C_6_-type carbides; meanwhile, high N tends to form nitrides, which suppress M_23_C_6_ precipitation, and other components have limited influence on M_23_C_6_. This conclusion aligns with the theory that carbide formation depends on the content of carbon and transition metal elements.

Finally, for PREN prediction, nitrogen (N), chromium (Cr) and molybdenum (Mo) are the dominant positive contributors, indicating that PREN is governed primarily by these corrosion-resistant elements. The remaining elements exert only minor effects, acting as weak modifiers over limited compositional ranges.

In summary, SHAP analyses across the six prediction tasks validate the reliability of the models and corroborate established alloy-design and phase-transformation theories, while revealing distinct, target-dependent elemental effects that differentiate the contributions of multiple elements to the predicted microstructural and performance metrics.

These SHAP patterns are consistent with the established metallurgical understanding of HNASS and allow each ML prediction to be decomposed into contributions from individual elements, thereby providing a physically interpretable link between composition, phase stability descriptors and corrosion resistance.

### 3.4. Decision Variables and Value Ranges

#### 3.4.1. Model Validation

To enable collaborative optimization of multiple performance indicators in alloy composition design, we formulate a six-objective optimization problem. The decision variables are the mass fractions of seven principal elements—C, N, Cr, Mn, Mo, Ni, and Si—within the prescribed composition bounds. The objective vector comprises the predicted values of six targets: the δ-ferrite temperature range, the freezing range, the Cr_2_N precipitation temperature, the σ-phase precipitation temperature, the M_23_C_6_ precipitation temperature and PREN. Consistent with design goals, the first five objectives are minimized and PREN is maximized. Each objective is evaluated by a surrogate model based on RF regression trained in the preceding section.

#### 3.4.2. Optimize Objective Function

The optimization objective consists of six performance indicators, denoted, respectively, as follows:

(5) $$f\left(X\right)=\left(f1\left(X\right),f2\left(X\right),f3\left(X\right),f4\left(X\right),f5\left(X\right),f6\left(X\right)\right)$$

This multi-objective optimization design aims to simultaneously reduce the precipitation risk of harmful phases (σ-phase, M_23_C_6_, etc.) and significantly improve pitting resistance. The resulting alloy composition system corresponds to high-nitrogen, low-carbon super-austenitic/super-duplex stainless steel, capable of achieving excellent corrosion resistance and structural stability in high-chloride and highly corrosive environments, among others (Table 5).

The corresponding target orientation is set to [1,1,1,1,1,−1], where “1” indicates a minimization objective and “−1” indicates a maximization objective.

#### 3.4.3. Optimization Algorithms and Parameter Settings

In order to simultaneously optimize the six conflicting design objectives considered in this work—minimizing the δ-ferrite temperature range, the freezing range and the precipitation/dissolution temperatures of Cr_2_N, σ-phase and M_23_C_6_, while maximizing PREN—the non-dominated sorting genetic algorithm III (NSGA-III) is employed to explore the composition space spanned by C, N, Cr, Mn, Mo, Ni and Si. NSGA-III is an improved version of the widely used NSGA-II algorithm and is specifically designed for many-objective optimization problems involving more than three objectives [59,60]. By combining classical non-dominated sorting with a set of predefined reference points in the objective space, NSGA-III preserves population diversity and yields a well-distributed approximation to the Pareto front even in six-dimensional objective space. These characteristics make NSGA-III particularly suitable for the present stainless steel design problem, where complex trade-offs between phase-stability descriptors and pitting-resistance must be captured quantitatively before applying the subsequent TOPSIS-based ranking.

**Figure 8:**
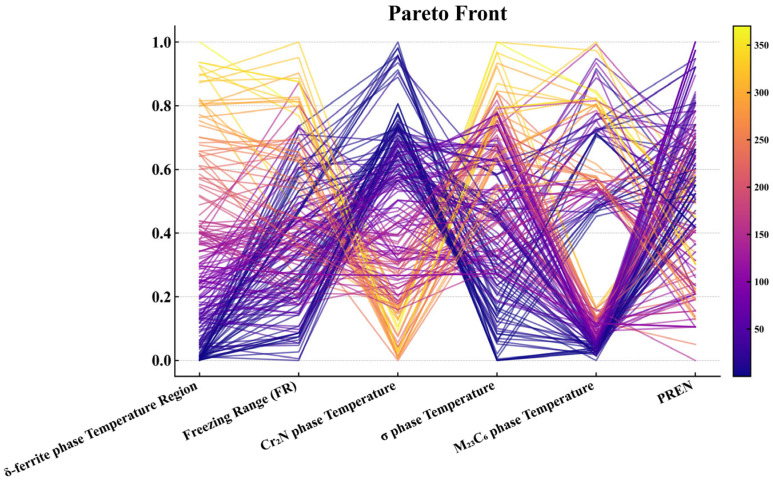
Parallel coordinate plot showing six optimized objectives from the MOEA, with red lines representing Pareto-optimal solutions.

As illustrated in Figure 8, the NSGA-III Pareto front is visualized using a parallel-coordinates plot in which all six objectives are scaled to [0,1] (lower values indicate better performance for the five temperature-related objectives, whereas higher values are preferred for PREN). Several consistent patterns can be observed. First, many trajectories combine a low δ-ferrite temperature range with a high PREN, indicating a favorable synergy between suppressing δ-ferrite and enhancing corrosion resistance. Second, high-PREN solutions frequently coincide with elevated Cr_2_N precipitation temperature and, to a lesser extent, higher σ-phase precipitation temperature, revealing an inherent trade-off between corrosion resistance and the tendency to form nitrides and intermetallics. Third, the M_23_C_6_ precipitation temperature can often be reduced concurrently with the σ-phase precipitation temperature, whereas the freezing range remains a bottleneck: solutions that strongly minimize the freezing range tend to exhibit only moderate PREN or an increase in at least one precipitation temperature. Overall, the Pareto front exhibits clear “knee” regions characterized by low δ-ferrite range, low-to-moderate σ and M_23_C_6_ precipitation temperatures, an acceptable freezing range and high PREN; these compromise solutions were subsequently prioritized in the TOPSIS-based ranking step.

### 3.5. Results Screening and Evaluation

After optimization, the non-dominated solutions form the Pareto front. To select representative candidates, we apply TOPSIS for comprehensive ranking. The six objective values for each solution are first normalized to a common scale and assigned equal weights of one. We then define an ideal best point with maximum PREN and minimum values for the five temperature-related objectives and an ideal worst point with the opposite attributes. For every solution, Euclidean distances to these two reference points are computed and converted into a relative closeness score, where a larger score indicates a better overall compromise. The highest-scoring solutions are retained for subsequent analysis and validation.

In this work, TOPSIS is applied to rank the Pareto-optimal solutions obtained from NSGA-III. First, all six objectives are normalized across the Pareto set to remove scale effects, with the five temperature-related descriptors treated as cost-type criteria (to be minimized) and PREN treated as a benefit-type criterion (to be maximized). The ideal best point in the normalized objective space is then constructed by taking the minimum values for all temperature-related objectives and the maximum value for PREN, whereas the ideal worst point is constructed by taking the maximum temperatures and the minimum PREN. For each candidate alloy, the Euclidean distances to the ideal best and ideal worst points are computed, and a relative closeness coefficient is obtained as the ratio between the distance to the worst point and the sum of both distances. A higher closeness coefficient therefore indicates a more favorable trade-off among the six objectives. In this first demonstration, equal weights are assigned to all objectives to avoid imposing subjective preferences; alternative weightings can be introduced in future work to emphasize specific design priorities (e.g., corrosion resistance versus phase-stability margins).

### 3.6. Optimization Results

The optimization results show that the TOPSIS relative closeness of the top ten candidates exceeds 0.82, indicating well-balanced performance across objectives. The top-ranked composition—C 0.01, N 0.80, Cr 18.0, Mn 19.5, Mo 1.80, Ni 3.90, Si 0.20 wt.%—achieves a closeness of 0.922 and yields the following predictions: δ-ferrite temperature range ≈ 0 °C, freezing range ≈ 155.0 °C, Cr_2_N precipitation temperature ≈ 1034.8 °C, σ-phase precipitation temperature ≈ 884.3 °C, M_23_C_6_ precipitation temperature ≈ 689.1 °C and PREN ≈ 36.74 (dimensionless). Other high-ranking solutions (ranks 2–5) present similarly credible trade-offs between PREN and the precipitation temperatures, confirming the effectiveness of NSGA-III combined with TOPSIS and the robustness of the surrogate-based optimization workflow.

In summary, multi-objective optimization followed by TOPSIS ranking produced a Pareto set within the prescribed bounds and identified composition candidates with superior trade-offs among objectives. These outcomes provide a quantitative basis for subsequent alloy design and performance tailoring.

**Figure 9:**
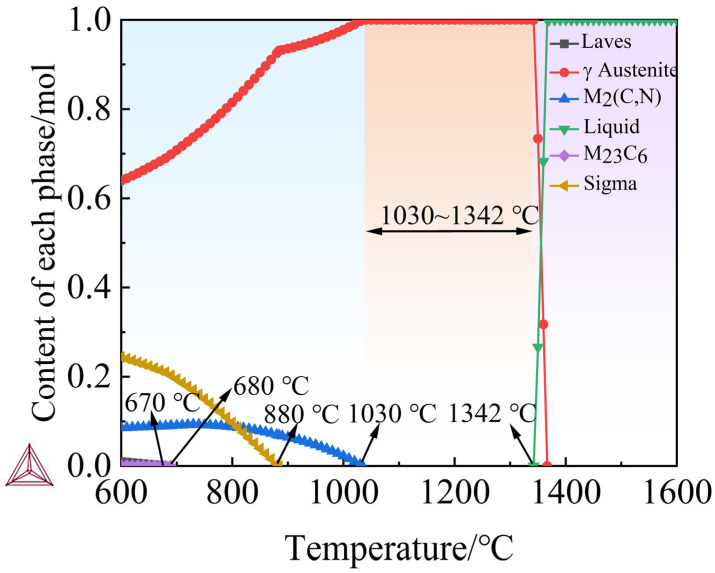
Equilibrium phase fractions of the designed high-nitrogen austenitic stainless steel (Fe–18.0Cr–19.5Mn–1.8Mo–3.9Ni–0.2Si–0.01C–0.8N wt.%) calculated using the CALPHAD method with the TCFE11 database.

Figure 9 presents the calculated equilibrium phase evolution for the optimized high-nitrogen austenitic stainless steel composition Fe–18.0Cr–19.5Mn–1.80Mo–3.90Ni–0.20Si–0.01C–0.80N (wt.%), obtained using the Calculation of Phase Diagrams (CALPHAD) approach with the TCFE13 database. The results show that austenite (γ) is thermodynamically stable over a broad interval of approximately 950–1400 °C, constituting nearly 100 wt.% of the solid prior to melting. The lack of an appreciable δ-ferrite field within this range indicates that the designed chemistry effectively stabilizes the austenitic matrix and suppresses δ-ferrite formation, which supports the targeted combination of high toughness and corrosion resistance.

At lower temperatures (<900 °C), small fractions of secondary phases—M_2_(C,N), M_23_C_6_ carbides and σ—are predicted. The M_2_(C,N) phase is stable over approximately 900–700 °C, consistent with the formation of Cr- and Mo-bearing carbo-nitrides that can contribute to precipitation strengthening. The σ-phase fraction remains limited (<5 wt.%), indicating a relatively low risk of brittle intermetallic embrittlement. A minor amount of Laves phase is also predicted below ~800 °C, which is typical for Mo-bearing austenitic steels. Crucially, all secondary phases dissolve completely above ~950 °C, enabling a homogeneous single-phase austenite during solution treatment and providing a favorable starting point for subsequent thermomechanical processing and mechanical performance.

Overall, thermodynamic simulations confirm that the machine learning-optimized composition achieves a balanced combination of austenite stability and controlled precipitation. This balance is essential for realizing the targeted synergy of high strength, ductility, and corrosion resistance in high-nitrogen austenitic stainless steels.

**Figure 10:**
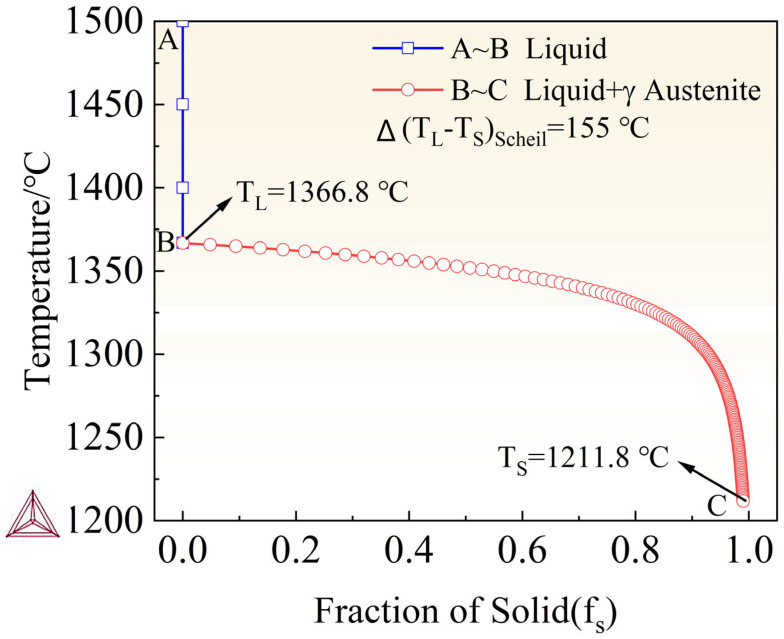
Scheil–Gulliver solidification path of HNASS. A–B: liquid; B–C: liquid + γ$T_{L}=1366.8^{\circ }C$, $T_{S}=1211.8^{\circ }C$; $\Delta T_{\mathrm{Scheil}}=155^{\circ }C$.

As shown in Figure 10, the Scheil–Gulliver non-equilibrium path of the high-nitrogen austenitic stainless steel exhibits primary austenite solidification (L → L + γ → γ), which simplifies transformation sequences and promotes reproducible, fully austenitic microstructures. The well-defined liquidus and solidus provide a clear freezing range that can be directly used to set process windows (e.g., preheat level, linear energy, scan speed) and to calibrate thermal–metallurgical simulations. Austenite-first solidification also helps retain nitrogen in the matrix, supporting higher PREN and good toughness by avoiding sequestration of N into δ-ferrite. Moreover, the smooth, monotonic $f_{s}$–T evolution and distinct landmarks (nucleation onset, coherency/coalescence intervals) yield clean, ML-ready descriptors, facilitating data-driven multi-objective optimization that links composition, processing, and properties. Overall, the figure offers actionable guidance for process design while preserving the intrinsic advantages of HNASS.

## 4. Conclusions

We constructed a six-objective optimization framework in which the mass fractions of seven principal elements (C, N, Cr, Mn, Mo, Ni, Si) serve as decision variables. The objectives are to minimize the δ-ferrite temperature range, the freezing range, the Cr_2_N precipitation temperature, the σ-phase precipitation temperature and the M_23_C_6_ precipitation temperature, while maximizing PREN. Using a random forest surrogate trained on thermodynamics-derived data, the optimized composition reduces the propensity for harmful precipitates (notably σ and M_23_C_6_), narrows the freezing range and thereby mitigates segregation and hot-cracking susceptibility, improving microstructural stability during welding and casting. While maintaining mechanical properties, the optimized alloy exhibits a substantially increased PREN; the resulting pitting resistance approaches or exceeds that of super-austenitic and super-duplex stainless steels, indicating suitability for long-term service in chloride-rich and strongly corrosive environments such as marine engineering, seawater desalination and chemical processing. This multi-objective strategy provides a quantitative basis and a transferable methodology for the compositional design of high-performance, corrosion-resistant stainless steels. Although the RF model achieves very high R^2^ values and low RMSE with respect to the CALPHAD data, this does not automatically guarantee agreement with experimental measurements. To mitigate overfitting at the surrogate level, we employed an 80–20 train–test split with fixed random seeds, three-fold cross-validation during hyperparameter optimization and controlled tree complexity. The close agreement between training and testing metrics (Table 3 and Table 4) and the absence of systematic divergence in the parity plots (Figure 4 and Figure 5) suggest that variance-driven overfitting is limited. Nevertheless, the overall predictive fidelity remains bound by the accuracy of the thermodynamic database, and experimental validation of the optimized compositions is required before industrial deployment. By construction, the six target descriptors in this work are outputs of equilibrium and Scheil CALPHAD calculations. The RF and XGBoost models are therefore trained to reproduce these thermodynamic quantities, and the high R^2^ values together with the narrow residual distributions indicate that the surrogates closely match the CALPHAD predictions without obvious systematic deviations in specific temperature ranges. The discrepancy between the present predictions and actual experimental behavior is thus primarily governed by the fidelity of the underlying thermodynamic database. Future work will focus on benchmarking representative optimized compositions against experimental phase-boundary and precipitation data to quantify these deviations more precisely.

Practical manufacturability considerations are partially embedded in the present framework through the choice of composition ranges and thermodynamic filters: the ranges of C, N, Cr, Mn, Mo, Ni and Si are centered on commercial HNASS and reported alloys, and compositions with excessive δ-ferrite, σ-phase or high-temperature carbides are removed, which indirectly promotes weldability and microstructural robustness. However, explicit cost metrics and process-route-specific constraints (e.g., nitrogen solubility margins for pressurized melting or processability windows for additive manufacturing) are not included as separate objectives or constraints in the current NSGA-III formulation. Future extensions of the framework could incorporate such quantities directly, enabling truly manufacturing-aware multi-objective optimization.

In future work, first-principle calculations will be integrated with multi-source experimental datasets to further improve the predictive accuracy of the models. Building on this, systematic experimental validation and service-performance evaluation will be conducted, with emphasis on the synergistic effects of thermomechanical processing routes and heat-treatment schedules on the freezing range, microstructural stability, and corrosion behavior. In parallel, the multi-objective optimization framework will be extended to a broader set of stainless steel systems and other high-performance alloys, enabling coordinated improvements in mechanical properties, corrosion resistance, microstructural stability, and processability.

The present study is purely computational and does not yet include experimental validation of the optimized compositions. Planned future work will synthesize representative Pareto-optimal alloys, followed by microstructural and corrosion characterization, in order to benchmark both the CALPHAD predictions and the ML-based optimization against experimental behavior.

## Figures and Tables

**Table 1:** Parameters used for the development of the compositional space in this study.

Elements (wt.%)	Elemental Content	Step Length
**N (0.55–0.80)**	0.55/0.60/0.65/0.70/0.75/0.80	0.05
**Mo (1.8–2.6)**	1.8/2.0/2.2/2.4/2.6	0.2
**Cr (18–19)**	18.0/18.2/18.4/18.6/18.8/19.0	0.2
**Ni (3.5–4.5)**	3.5/3.7/3.9/4.1/4.3/4.5	0.2
**Mn (19.5–22.5)**	19.5/20.0/20.5/21.0/21.5/22.0/22.5	0.5
**Si (0.2–0.4)**	0.2/0.4	0.2
**C (0.01–0.04)**	0.01/0.02/0.03/0.04	0.1
**Total of Alloys’ Candidates**	the total number of combinations: 6 × 5 × 6 × 6 × 7 × 2 × 4 = 60,480	**60,480**

**Table 2:** Feature description.

Name	Description
δ-ferrite phase temperature	Ferritic phase that controls the solidification mode and strongly affects toughness and hot-cracking susceptibility in high-nitrogen austenitic stainless steels.
Cr_2_N phase temperature	Chromium nitride precipitate that consumes Cr and N from austenite, thereby degrading pitting resistance and lowering toughness, especially along grain boundaries.
σ-phase temperature	Cr- and Mo-rich intermetallic phase that causes severe embrittlement and depletes Cr and Mo from the matrix, reducing both toughness and localized corrosion resistance.
M_23_C_6_ phase temperature	Grain-boundary carbide that induces sensitization through local Cr depletion and simultaneously alters grain-boundary strength and creep resistance.
Freezing range	Temperature interval between the liquidus and the solidus; a larger freezing range promotes microsegregation, hinders feeding and increases the risk of solidification cracking.
PREN	Empirical pitting resistance equivalent number that quantifies resistance to localized corrosion in chloride-containing environments, with higher values indicating better performance.

**Table 3:** XGBoost model prediction metrics for each feature.

Type	Dataset Type	R^2^	MAE	RMSE
δ-ferrite phase temperature Region	Training set Test set	0.922 0.911	18.387 18.701	26.058 27.819
Freezing range	Training set Test set	0.917 0.916	4.309 4.311	5.231 5.249
Cr_2_N precipitation temperature	Training set Test set	0.953 0.929	4.904 4.931	5.972 6.024
σ-phase temperature	Training set Test set	0.930 0.929	4.446 4.508	5.659 5.732
M_23_C_6_ phase temperature	Training set Test set	0.974 0.974	6.792 6.758	8.204 8.164
PREN	Training set Test set	0.966 0.965	0.254 0.254	0.313 0.309

**Table 4:** Random forest model prediction metrics for each feature.

Type	Dataset Type	R^2^	MAE	RMSE
δ-ferrite phase temperature Region	Training set Test set	0.984 0.980	10.44 10.31	13.91 12.95
Freezing range	Training set Test set	0.986 0.98.	1.37 1.27	1.76 1.65
Cr_2_N precipitation temperature	Training set Test set	0.999 0.998	0.98 0.979	1.27 1.23
σ-phase temperature	Training set Test set	0.981 0.978	2.11 2.11	2.62 2.65
M_23_C_6_ phase temperature	Training set Test set	0.974 0.974	1.08 1.09	1.43 1.41
PREN	Training set Test set	0.999 0.999	0.003 0.003	0.004 0.004

**Table 5:** Performance metric range.

Number	Name	Value
f1	δ-ferrite phase temperature Region	Min
f2	Cr_2_N phase temperature	Min
f3	σ-phase temperature	Min
f4	M_23_C_6_ phase temperature	Min
f5	the freezing range	Min
f6	PREN	Max

## Data Availability

The original contributions presented in this study are included in the article. Further inquiries can be directed to the corresponding author.
